# Contribution of Minimally Invasive Bone Augmentation With PMMA Cement in Primary Fixation of Schatzker Type II Tibial Plateau Fractures

**DOI:** 10.3389/fbioe.2022.840052

**Published:** 2022-03-01

**Authors:** T. Vendeuvre, C. Koneazny, C. Brèque, P. Rigoard, M. Severyns, A. Germaneau

**Affiliations:** ^1^ Institut Pprime UPR 3346, CNRS, ISAE-ENSMA, Université de Poitiers, Poitiers, France; ^2^ Department of Orthopaedic Surgery and Traumatology, University Hospital, Poitiers, France; ^3^ PRISMATICS Lab, Department of Spine Surgery and Neuromodulation, University Hospital, Poitiers, France; ^4^ Department of Orthopaedic Surgery and Traumatology, University Hospital, Martinique, France

**Keywords:** tibial plateau fracture osteosynthesis, tuberoplasty, percutaneous osteosynthesis, injectable bone cement, optical methods, biomechanics

## Abstract

**Background:** The most common type of fracture of the lateral tibial plateau is the Schatzker type II split-depressed fracture. Minimally invasive surgery using balloon reduction appears to be very promising compared to the gold standard using a bone tamp. This surgery aims to have the best reduction and stabilization to benefit from an early passive and active rehabilitation to avoid stiffening and muscle wasting. Using a balloon for fracture reduction has allowed the use of semi-liquid Injectable Bone Cement (IBC) fillers. These fillers can be phosphocalcic or polymethyl methacrylate (PMMA). The latest recommendations on these IBCs in spinal surgery increasingly rule out phosphocalcic fillers because of their low mechanical strength.

**Questions/purposes:** 1) What is the mechanical influence of IBC filling (PMMA) regarding the split and depression components of a Schatzker type II fracture? 2) What is the mechanical influence of osteosynthesis regarding the split and depression components of a Schatzker type II fracture with or without PMMA filing in three different kinds of percutaneous fixations?

**Methods:** This biomechanical study was performed on 36 fresh frozen tibia/fibula specimens. Six groups were formed according to the type of percutaneous osteosynthesis or possible PMMA filling. Mechanical strength tests were carried out using a Unicompartmental Knee prosthesis and displacement components were measured on either side of the separation on the anterolateral facet by optical method.

**Results:** We found a significant difference between cementless and cemented osteosynthesis for depression fracture stabilization (difference −507.56N with 95% confidence interval [−904.17; −110.94] (*p*-value = 0.026)). The differences between the different types of osteosynthesis were not significant (*p*-value = 0.58). There was a significant difference between osteosynthesis without cement and osteosynthesis with cement on separation (difference −477.72N [−878.52; −76.93] (*p*-value = 0.03)). The differences between the different types of fixations were not significant regarding separation (*p*-value = 0.99).

**Conclusion:** PMMA cement significantly improves primary stability, regardless of the type of osteosynthesis for a Schatzker type II plateau fracture. Filling with PMMA cement during tuberoplasty seems to be a very promising strategy in association with percutaneous osteosynthesis to allow rapid recovery after surgery.

## Introduction

The most common type of fractures of the lateral tibial plateau is the Schatzker type II split-depressed fracture (Schatzker et al., 1979; Zeltser and Leopold, 2013). Minimally invasive surgery using balloon reduction appears to be very promising compared to the gold standard using a bone tamp (Ollivier et al., 2016; Doria et al., 2017). This surgery aims to have the best reduction and stabilization to benefit from an early passive and active rehabilitation to avoid stiffening and muscle wasting (Wang et al., 2018). The goal is also to achieve early weight-bearing for rapid recovery and minimize the risk of complications related to decubitus (Wang et al., 2018; Vendeuvre and Gayet, 2021). Using a balloon for fracture reduction has allowed the use of semi-liquid Injectable Bone Cement (IBC) fillers. These fillers can be phosphocalcic or polymethyl methacrylate (PMMA). The latest recommendations on these IBCs in spinal surgery increasingly rule out phosphocalcic fillers because of their low mechanical strength (Singh et al., 2019). PMMA is the injectable filler that offers the best mechanical properties in compression (Wittenberg et al., 1993; Kayanja et al., 2006). It is regularly used in orthopedic surgery for filling vertebral fractures or for arthroplasty (Lieberman and Reinhardt, 2003; Singh et al., 2019; Sebastian et al., 2020).

Minimally invasive plate osteosynthesis systems have been developed with a mindset similar to percutaneous screw fixation. These systems allow for optimal positioning of the screws under the depressed part of the fracture to have a mechanical strut while controlling the separation (Mauffrey et al., 2014). This use of minimally invasive plates is often referred to as MIPPO (Minimal Invasive Percutaneous Plating Osteosynthesis). Percutaneous screws are the most widely used because their ease of use and modern plating systems allow for optimized screw positioning and locking to the plate.

There are two main locking techniques for plating systems: direct locking by screw head threads or secondary locking by caps. These designs offer differences in the number of screws positioned in the epiphysis and the screw diameters. Our study compares the NCB^®^ percutaneous plate, which uses Caps locking, with the VA-LCP^®^ percutaneous plate, which uses screw head thread locking and fixation using percutaneous screw alone.

The main goal of this study was to compare different minimally invasive fracture fixations in association with balloon reduction using the tuberoplasty technique. We compared the primary structural strength of the fracture fixation regarding the separation and depression components. We also tested the mechanical influence of IBC filling on these three types of percutaneous fixations.

## Methods

### Preparation of the Parts

This biomechanical study was performed on 36 fresh frozen tibia/fibula specimens collected at the ABS Lab laboratory of the University of Poitiers (Ministry of Education and Research No. DC-2008-137). They were divided into six groups, each with six randomized specimens, homogeneous in age, sex, and laterality (Table 1).

**TABLE 1:** Distribution of anatomical parts according to age, sex, and laterality.

	G1	G2	G3	G4	G5	G6
Sex (M/F)	3/3	3/3	3/3	3/3	3/3	3/3
Laterality (R/L)	3/3	3/3	3/3	3/3	3/3	3/3
Age (Years ± DS)	85 ± 8.10	85 ± 8.1	78.8 ± 6.2	74.6 ± 11.4	77.3 ± 12.4	77.6 ± 12.1

In order to address the different issues, the six groups were formed as follows:

-Group 1 was reduced using a balloon and osteosynthesis with a Zimmer^®^ plate and filled with PMMA cement;
-Group 2 was reduced by balloon then osteosynthesis by Zimmer^®^ plate without filling;
-Group 3 was reduced by balloon then osteosynthesis by two Maconor^®^ screws with washers without filling;
-Group 4 was reduced by balloon then osteosynthesis by two Maconor^®^ screws with washers and PMMA cement filling;
-Group 5 was reduced with a balloon and then osteosynthesis with a Synthes^®^ plate and PMMA cement filling;
-Group 6 was reduced with a balloon and then osteosynthesis with a Synthes^®^ plate without filling.

Specimen preparation and fracture pattern were performed according to Vendeuvre et al. (Vendeuvre et al., 2018).

Specimens were prepared using the proximal tibia and fibula up to the middle diaphysis. They were fixed in a rigid polyurethane resin base (Allrim^®^) and stabilized in an anatomical position.

The Schatzker type II fracture pattern was made in a standardized and reproducible matter.

Post-fracture CT analysis was performed to confirm the Schatzker type II fracture. All fractures were validated, and, at this stage of the study, no specimens were excluded.

### Fracture Reduction and Fixation

Fractures were reduced by tuberoplasty with a 20-mm Medtronic Sofamor Expender 2 kyphoplasty^®^ balloon using an anterior MIS approach (Vendeuvre et al., 2013).

#### The Different Materials

• Percutaneous osteosynthesis

**FIGURE 1:**
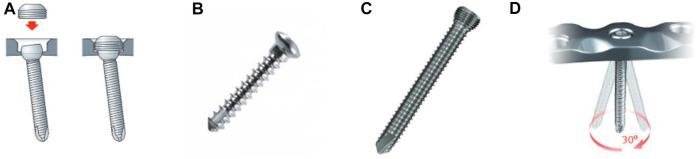
Three differents kind of screw fixation. **(A)** Caps used for locking screws in NCB Zimmer^®^ plates, **(B)** Maconor^®^ bicortical screw without plate, **(C)** Epiphysial screw with a 30° range in the Synthes^®^ plate, and **(D)** 30° variable axis for the tool locking system.

The choice of fixation techniques was: 1) percutaneous system, 2) placement of polyaxial screws. We then selected three types of osteosynthesis (Figure 1).

• Two Maconor (Howmedica^®^) bicortical screws, 4.5 mm in diameter, for groups 3 and 4 (Figure 1B).

Fracture fixation was performed in both groups using two 4.5 mm diameter Maconor (Howmedica^®^) bicortical screws and a 13 mm stainless steel washer. The trajectory was prepared with a 3.4-mm drill bit, and the screws were positioned with a lateral entry point located below the most distal part of the depression. The configuration of the osteosynthesis screws was the same for both groups.

• Zimmer^®^ NCB Proximal Tibia Plate + Screws for Groups 1 and 2 (Figure 1A).

In this study, we used a lateral anatomical plate compatible with a percutaneous minimally invasive ancillary. This plate has three 4.5 mm epiphyseal screws with spherical heads locked secondarily with caps (Figure 1A). Screws can be oriented in a 30° radius. The shortest plate, 132 mm long, was used, as well as three bi-cortical diaphyseal screws. The system allows the placement of polyaxial screws (30°) with subsequent locking of the screws and use of locking caps.

• Synthes®VA-LCP Proximal Tibial Plate 3.5 for Groups 5 and 6 (Figure 1C).

In this study, we chose a lateral anatomical plate that could be used with a percutaneous ancillary. Two models were available, allowing a choice between two radii of curvature (Small Bend and Large Bend) and adapting to the anatomy of each subject. This plate has four 3.5 mm diameter epiphyseal screws with self-locking threads. They are adjustable and can be positioned within a 30° radius (Figure 1D). We chose the shortest plate, 87 mm long, which was more than sufficient for our fracture model. Three bi-cortical screws were systematically positioned at the diaphyseal level.

• Medtronic^®^ balloon

We used a Medtronic Sofamor^®^ balloon kyphoplasty Xpander^®^ IBT 20 mm long and a capacity of 10 ccs or 350 PSI.

• PMMA cement

We chose to fill the gaps in groups 1, 4, and 6 with PMMA KyphX HV-R^®^ Kyphon^®^ cement, whose composition is as follows:

-Barium sulfate
-Benzoyl peroxide
-Methyl methacrylate
-N, N-dimethyl-p-toluidine
-Hydroquinone

The role of the cement is to increase the mechanical strength of the fixation with which it may be associated, thus allowing for early active rehabilitation and a faster return to weight-bearing. As shown in the management of spinal fractures, we did not use phosphocalcic cement, which has a lower mechanical strength (Blattert et al., 2009). In addition, a tibia has to bear a greater weight than a vertebra.

### Biomechanical Tests

**FIGURE 2:**
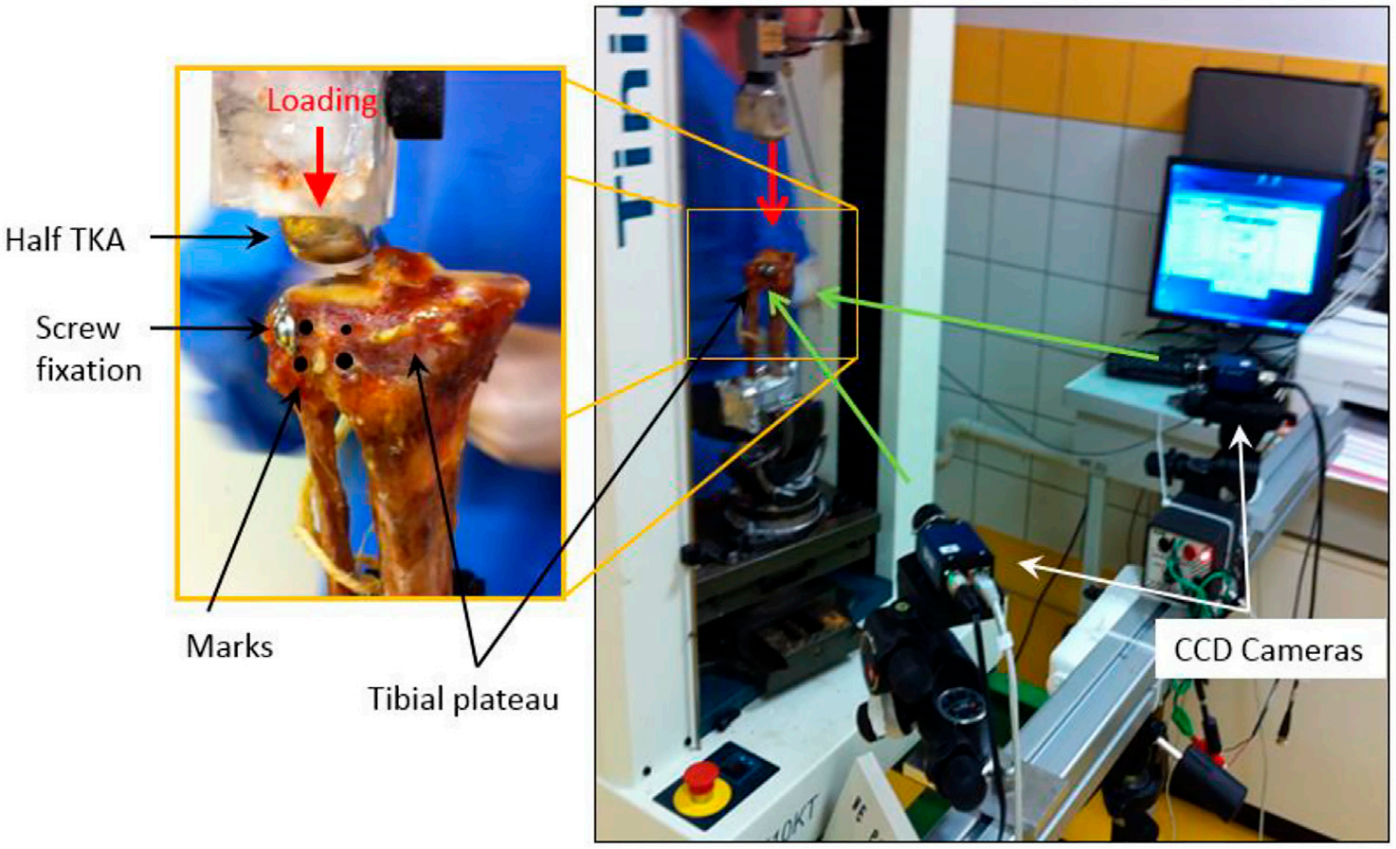
Mechanical strength tests were carried out using a Unicompartmental Knee prosthesis positioned on the lateral tibial plateau (Vendeuvre et al., 2018).

Mechanical strength tests were carried out using a Zimmer Unicompartmental Knee High Flex^®^ prosthesis, size 48 mm, positioned on the Tinius Olsen^®^ compression machine (able to provide up to 10kN loading), positioned on the lateral tibial plateau (Figure 2) (Vendeuvre et al., 2018).

The loading rate was set at 5 mm/min. The compression force on the tibial plateau was measured by a load cell (up to 10kN) located between the mobile span and the unicompartmental knee prosthesis. Four marks were placed on either side of the separation on the anterolateral facet of the specimen to measure the distance between the fracture line by 3D mark tracking. These four marks were used to characterize the separation of bone fragments after stabilization and during the loading. The depression fracture was characterized by measuring the displacement of the unicompartmental knee prosthesis according to loading.

The mark tracking allowed us to study the bone deformations in compression and separation as a function of the applied force and determine the force’s value necessary to cause plastic deformation of the upper end of the tibia, corresponding to the assembly’s failure.

The characterization of the mechanical response of the stabilization systems was carried out by identifying the limiting force **Fe** determining the transition from linear response to a non-linear response corresponding to the transition from an elastic bone deformation to plastic deformation.

### Statistical Analysis

All measured data was computerized and analyzed using R 3.0.1^**TM**^ statistical software. Comparisons between groups (for quantitative mechanical parameters) were performed using a non-parametric Wilcoxon Man Whitney test. A Tukey test was also used to compare the groups. Finally, the influence of PMMA was calculated using a multivariate analysis of variance (MANOVA) with two factors: osteosynthesis and PMMA.

## Results

For each group sample, we obtained a value of elastic limit on the depression and separation components.

Then, we studied the influence of the different types of osteosynthesis and PMMA filling on the Schatzker II fractures.

### Results on the Depression Fracture

**FIGURE 3:**
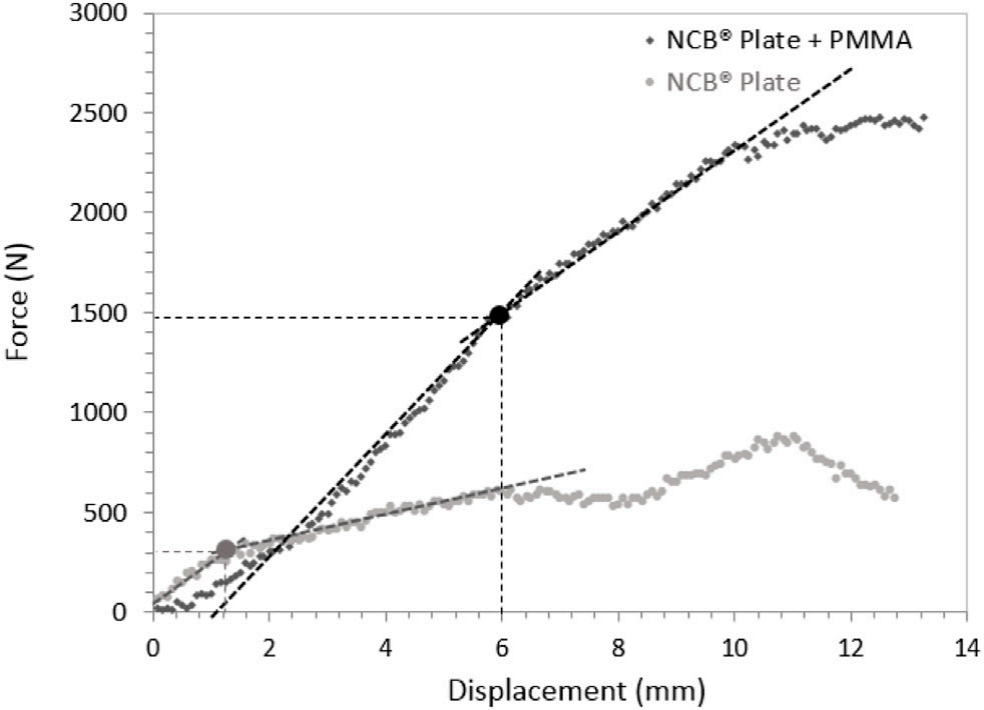
Example of load-displacement curves for depression analysis for specimens stabilized with NCB^®^ plates, with and without PMMA filling.

Figure 3 shows the load vs. displacement (of the knee prosthesis) curves for the depression component for two representative specimens of the NCB^®^ plate group (with and without PMMA filling). This graph illustrates the mechanical response of the fracture fixation under compressive loading with a specimen that a linear behavior can approximate. Elastic limit forces are measured at the transition between the linear and the other part of the curve.

We found a significant difference between cementless and cemented osteosynthesis for depression fracture stabilization (difference −507.56 N with 95% confidence interval [−904.17; −110.94] *p*-value = 0.026). The differences between the different types of osteosynthesis were not significant (*p*-value = 0.58).

A two-factor MANOVA analysis showed that only the addition of cement had an influence on the depression (*p* = 0.013) and that neither the type of synthesis nor the interaction between the type of osteosynthesis (*p* = 0.43) and cement influenced the depression (*p* = 0.82).

### Results on Separation

There was a significant difference on the separation component (measured from the marks disposed on either side of the fracture) between osteosynthesis without cement and osteosynthesis with cement on separation (difference −477.72N with 95% confidence interval [−878.52; −76.93] *p*-value = 0.03). The differences between the different types of fixations were not significant regarding separation (*p*-value = 0.99) (Tables 2, 3).

**TABLE 2:** Statistical results of the comparisons of the measured limiting force values between the different groups (Wilcoxon Mann Whitney test).

Comparison of the different assemblies (2 by 2)	Depression *p*-value	Separation *p*-value
Screw:Cement/Plate VA-LCP:Cement	0.9999	0.9220
Plate NCB:Cement/Plate VA-LCP:Cement	0.9071	0.8166
Plate NCB:Cement/Screw:Cement	0.8097	0.9998
Plate VA-LCP:No Cement/Plate VA-LCP:Cement	0.6547	0.9999
Screw:No Cement/Screw:Cement	0.8935	0.2333
Plate NCB:No Cement/Plate NCB:Cement	0.3942	0.4765
Screw:No Cement/Plate VA-LCP:No Cement	0.9998	0.8198
Plate NCB:No Cement/Plate VA-LCP:No Cement	0.9907	0.9959
Plate NCB:No Cement/Screw:No Cement	0.9991	0.9763

**TABLE 3:** Comparison of the measured limiting forces between the different fixations with and without PMMA cement regarding separation and depression (Tukey test).

Groups	Depression			Separation		
	Mean (N)	STD (N)	*p*-value	Mean (N)	STD (N)	*p*-value
G1−NCB^®^ + PMMA	1864.7	947.4	0.394	1519.7	894.0	0.476
G2−NCB^®^	1209.3	491.2		897.7	442.5	
G3−Screw	1088.7	397.2	0.893	645.8	411.9	0.233
G4−Screw + PMMA	1444.8	222.0		1430.7	273.0	
G5−VA-LCP^®^	1010.0	525.3	0.654	1068.5	565.5	0.999
G6−VA-LCP^®^ + PMMA	1521.2	644.1		1094.8	757.3	

**FIGURE 4:**
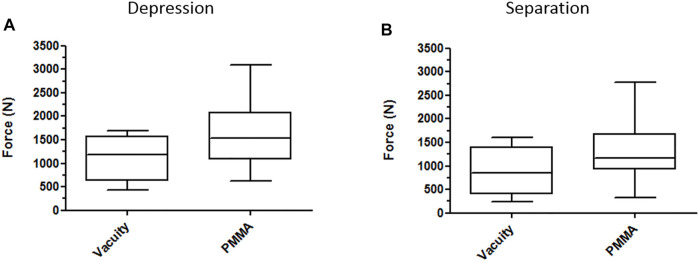
Box plots on the contribution of PMMA in the stabilization of depression **(A)** and separation **(B)** components on all types of fixations.

A two-factor MANOVA analysis showed that only the addition of cement had an influence on separation (*p* = 0.023) and that neither the type of synthesis nor the interaction between the type of osteosynthesis (*p* = 0.77) and cement influenced separation (*p* = 0.28) (Figure 4).

## Discussion

The most important finding of this study was that PMMA cement significantly improves the primary stability in case of Schatzker type II tibial plateau fractures. And the multivariate analysis of variance confirms that PMMA, independent of the type of osteosynthesis used, strengthens the fixation.

### Mechanical Influence of PMMA Filling

This fracture pattern is the most common tibial plateau fracture, and minimally invasive surgical management is increasingly used (Vendeuvre and Gayet, 2021). The balloon reduction technique has already been studied without filling and provides better mechanical stabilization than the bone tamp technique (Vendeuvre et al., 2018). Using a balloon to reduce tibial plateau fractures offers the possibility of using a semi-liquid filler such as PMMA. PMMA has also previously shown efficacy on pull-out tests on pedicle screws. A previous biomechanical study with a finite element method has shown that the cement filling of the tibial depression fracture may increase implant stability and decrease the loss of depression reduction, while the presence of the cement in the healed model renders the load distribution uniform (Belaid et al., 2018). Different clinical studies also did not report loss of reduction (Gosling et al., 2005; Evangelopoulos et al., 2010; Ollivier et al., 2016; Cuzzocrea et al., 2018). When reducing the fracture, the balloon compacts the cancellous bone and thus avoids the risk of cement leakage. The semi-liquid injection is then secured. Due to the proximity to the cartilage, no cement leakage can be allowed because polymerization is done at 80°C. Polymerization at 90°C can induce a risk of cartilage, skin, nerve, and vascular burns (Verlaan et al., 2003). However, bone vitality appears to remain intact according to SPECT/CT analysis after cement-augmented balloon tibioplasty (Jentzsch et al., 2015) when technical principles and indications are strictly respected (Mauffrey et al., 2014; Vendeuvre and Gayet, 2021).

### Mechanical Influence of Osteosynthesis Regarding the Split and Depression Components

The Schatzker fracture has two components: separation and depression (Schatzker et al., 1979; Zeltser and Leopold, 2013). The purpose of fixation for these fractures is to control these two parameters. Therefore, screws must be positioned below the depression to control the separation. It is also necessary to increase the contact to the cortical bone by using washers or plates to decrease the stress for better control of the separation (Mauffrey et al., 2014). The new plating systems meet this requirement: screws that can be angled (30°) to position themselves under the bone recess and an anatomical shape to fit the shape of the bone best and thus increase the external contact surface for separation control. The NCB^®^ Zimmer plate offers a medium curvature, and the Synthes VA-LCP^®^ plate offers the advantage of two possible curvatures to accommodate individual variability best. We used the latest minimally invasive locked plates, therefore being consistent with Boisrenoult’s study (Boisrenoult et al., 2000), which showed no difference between non-locked plates and screw fixation. At the time of this previous study, it was impossible to use MIPPO mini-invasive plates; therefore, mini-invasive reduction fixation under arthroscopy was done using only screws. Nowadays, plating systems have changed and been perfected, using locked screws, but the conclusion remains the same. Our study confirms the absence of significant difference between the three kinds of percutaneous osteosynthesis.

The fixation system one uses is a matter of habit and depends on its possibility to stabilize the reduced fracture. Indeed, a fracture in which separation pattern is dominant will be more easily reduced and stabilized using a plate, which is not the case if the depression is preponderant.

We created a reproducible cadaveric Schatzker type II fracture for this study (Belaid et al., 2018). However, many variations for this type of fracture can be seen, the separation can be preponderant, or the depression can be. If the separation is predominant, then its control can be much simpler with an anatomically shaped plate with a console-type fixation on the reduction obtained by ligamentotaxis. If the depression pattern is predominant, screw fixation is simpler and sufficient (Vendeuvre and Gayet, 2021).

### Impact on Postoperative Rehabilitation

Results from our study allow for optimistic outcomes regarding postoperative rehabilitation and may lead to early return to full weight-bearing. In 1970, Morrison observed that walking requires 200–300% of the bodyweight (a percentage that increases when standing and going down the stairs) (Morrison, 1970), corresponding to 1570–2360N for an 80 kg patient. In our study, we observed fixation failure with fracture displacement at an average of 1609.6 N when fixation was done after balloon reduction with PMMA filling, and 1102.3 N in the group without filling. Patients weighing less than 80 kg could thus resume full weight-bearing, protected by two crutches, immediately after surgery.

### Strengths of the Study

The use of mark tracking technique highlights in an original way, without degrading the material, the stabilization of the split component of the fracture. We noticed that the elastic limit value reached the fastest for each group was the value for the split element. Fixation failure regarding split-depressed fractures would therefore be primarily due to the split component. Therefore, it seems necessary that this fracture component is correctly studied. The study of the load impact from the unicompartmental knee prosthesis shows the effect on controlling the depression element of the fracture. With a confidence interval of 95%, we increase by 500 N the bearable load allowed by PMMA. This mechanical contribution is beneficial for both components of these fractures, depression, and separation.

The high number of specimens used in this study with a reproducible and validated fracture model.

### Study Limitations

The patient’s age was not representative of the young, non-osteoporotic subject; there were few specimens per subgroups of 6. Our study tended towards the superiority of the MIS plates, which was not significant because of this small number of specimens. The main limitations of our study may lie in the lack of dynamic cyclic testing, which is particularly indicative of *in vivo* conditions, and the fact that we used a unicompartmental and not a total knee prosthesis to apply the load. That said, *in vivo* conditions were not sought in this study, and using a total knee prosthesis would not have allowed for an even distribution of compressive forces on the lateral tibial plateau. In future works, dynamic cyclic testing will be considered for the development of a specific loading setup combining experimental procedures with a finite element modeling (Aubert et al., 2021).

## Conclusion

In a Schatzker type II plateau fracture, the use of a balloon for fracture reduction has been shown to increase the primary stability of the fixation when compared to bone stamp reduction alone. In addition, the balloon allows the use of a semi-liquid filler such as injectable PMMA bone cement. PMMA cement significantly improves primary stability, regardless of the type of osteosynthesis. The choice regarding which fixation system to use can therefore be left to the surgeons’ appreciation. Filling with PMMA cement seems to be a very promising strategy in association with percutaneous osteosynthesis to allow rapid recovery after surgery.

## Data Availability

The raw data supporting the conclusions of this article will be made available by the authors, without undue reservation.
